# It’s Not the Model, It’s the Way You Use It: Exploratory Early Health Economics Amid Complexity Comment on "Problems and Promises of Health Technologies: The Role of Early Health Economic Modelling"

**DOI:** 10.15171/ijhpm.2020.04

**Published:** 2020-01-18

**Authors:** Andrew Partington, Jonathan Karnon

**Affiliations:** ^1^Flinders Health and Medical Research Institute, Flinders University, Adelaide, SA, Australia.; ^2^Macquarie University Centre for the Health Economy, Macquarie University, Macquarie Park, NSW, Australia.

**Keywords:** Economic Evaluation, Health Systems, Complexity, Decision-Making, Early Assessment

## Abstract

In a review recently published in this journal, Grutters et al outline the scope and impact of their early health economic modelling of healthcare innovations. Their reflections shed light on ways that health economists can shift-away from traditional reimbursement decision-support, towards a broader role of facilitating the exploration of existing care pathways, and the design of options to implement or discontinue healthcare services. This is a crucial role in organisations that face constant pressure to react and adapt with changes to their existing service configurations, but where there may exist significant disagreement and uncertainty on the extent to which change is warranted. Such dynamics are known to create complex implementation environments, where changes risk being poorly implemented or fail to be sustained. In this commentary, we extend the discussion by Grutters et al on early health economic modelling, to the evaluation of complex interventions and systems. We highlight how early health economic modelling can contribute to a participatory approach for ongoing learning and development within healthcare organisations.

## Background

In presenting their experience in the use of early health economic modelling, Grutters et al describe the *“… shift away from the traditional use of health economic modelling … towards exploring what is needed for a technology to provide most value for money.”*^1^ This concluding message echoes that of a recent review by Scotland and Bryan, who similarly sought to challenge our traditional “technology evaluator” approach and advocated for an updated “searchers for efficiency” role.^2^ Health economists working in such a role are tasked with shifting the focus of decision-makers away from binary go/no-go decisions, to instead developing a better and shared understanding of the conditions under which interventions may represent value-for-money.

This is not a simple task. For economic evidence and evaluations to have traction in such a way, they must generate and present information that is both accessible and understandable to stakeholders with diverse professional backgrounds; and acceptable in terms of its relevance to stakeholders’ local setting and political sensitivity.^3^ This seems familiar to Grutters et al, who mention their efforts with model validation, and stress the importance of addressing local budget impacts.

The following sections of this commentary describe the complexities of interventions to improve healthcare services, and of the systems in which they are implemented. Within this context, we then outline the usefulness of early health economic modelling; and highlight important methodological considerations regarding uncertainty.

## Complex Interventions and Systems

As discussed by Sutton et al, there is an emergence of complex interventions, that exhibit diffuse, variable and broader spillover effects.^4^ Diffuse effects particularly arise when interventions are delivered at a patient group, rather than individual level and influence multiple care processes. Examples include delivery models that shift tasks between practitioners within different physical locations, perhaps facilitated by information communication technologies. The effects of such interventions may also vary across organisational settings, due to differences in existing workforce capabilities or physical space, but also due to variations in the clinical and sociodemographic characteristics of patients, which may impact their access to informal care at home or smart phones. Spillovers occur where the introduction of an intervention influences referral patterns, patient flows and the utilisation of other substitute or complimentary services. The competition for constrained resources or capacity can occur between services that are adjacent and down-stream to the target of a new intervention and represent additional financial impacts that may warrant inclusion within health economic models.

Grutters et al identified dependency on organisational setting and the presence of spillover effects as barriers, noting *“[o]ne specific barrier that we encountered several times was that an innovation would result in reallocating care from one clinical specialty to another.”*^1^ Such “*… broader criteria were not quantified*” in their early health economic modelling, but they noted that these effects *“… may limit the commercial viability of an innovation.”*^1^

The health services into which complex interventions are implemented are themselves, complex-adaptive systems. Such systems are characterised by interacting stakeholders, who actively pre-empt, adapt and influence the technical and social developments associated with an intervention. Different stakeholders may adapt to differing extents, often with alternate goals, which invariably impact on the success of implementation.^5,6^ Key distinctions between simple implementation settings and more complex (sometimes bordering on chaotic) settings are relatively high levels of disagreement amongst stakeholders regarding potential options (eg, options for targeting and sequencing interventions), and stakeholders’ conceptualisation of the magnitude and uncertainty of the expected effects of different options.^7^

## Participatory Modelling for Iterative Development and Implementation

Grutters et al experience that early health economic modelling rarely informs a binary ‘go/no go’ decision, also applies to interventions that are more complex, or are to be implemented in complex systems. Instead, the more relevant decision focus may be a ‘not yet’ or ‘yes, but (with conditions)’ decision, using economic modelling as an iterative and ongoing process. This requires the encouragement of nuanced deliberation, applying participatory methods that help stakeholders understand both the possibilities, about which they may be unaware or disagree; and the probabilities, around which there is an uncertainty of effects.^8^

This reframing of the decision problem denotes the distinct role of early heath economic modelling to support a formative process that shapes the development and tailoring of interventions. The promotion of iterative economic modelling for such support is nothing new,^9^ but its application has been limited. However, there appears to be renewed encouragement and receptiveness within health services to foster “learning systems,”^10^ whereby the complexities of a service and intervention are explicitly represented, and explored in an iterative process. Such a process facilitates the engagement of diverse stakeholders in the development of interventions through a common language and framework.^5^

Grutters et al reported that their early health economic modelling helped to identify and pre-empt challenges; interestingly though however, only one of the 33 assessments resulted in further research into the implementation setting (ie, usual care).^1^ In the context of complex systems, there may be benefit in also modelling the effectiveness of existing technologies *in situ*, to better understand aspects of current care pathways with the greatest capacity for improvement, to inform the design of new delivery models.

This corresponds to the notion that it is not necessarily the model itself that is informative, but rather the modelling process. Indeed, the interim National Institute for Health and Care Excellence (NICE) guidance on service guidelines suggests this: *“Even if a fully modelled analysis is not possible, there is value in the process of development, as it will help to structure Committee discussions.”*^11^

## Uncertainty and Formative Evaluations

Love-Koh notes in his commentary on Grutters et al notes that probabilistic sensitivity analyses is the gold standard for representing overall uncertainty and valuing the collection of further information.^12^ Grutters et al make a fair point that *“… for an audience without expertise in economic evaluation, we feel that scenario and threshold analyses, as well as deterministic sensitivity analyses … are most informative in providing insight.”*^1^ Further, they highlight the real risks of *“pseudo-certainty of the results.”*^1^ There is a need to guard against ‘garbage in, garbage out’ modelling based on data that we know to be fragmented, poorly linked with long-term outcomes, and inconsistently/sparsely populated.^13^

Informed, explicit elicitation of a models’ input parameters can be scientific and a means to illuminating the extent of uncertainty and providing a framework for exploring underlying assumptions, to be contested and updated with additional evidence.^14^ In addition to representing input parameter uncertainty and the value of ongoing data collection, the need to represent structural uncertainty may be more acute for early health economic modelling than at later stages when there may be greater certainty on the validity of a single model structure.^15^ Methods for the elicitation of information from relevant experts continue to improve and these methods are likely to strengthen and improve the value of early health economic modelling.

## The Take-Home Message

Grutters et al reported on the use of early health economic modelling, noting the rarity of ‘no go’ decisions and advocating for an exploratory role for health economists.^1^ This highlights the usefulness of an iterative health economic modelling process, rather than the model itself, to improve understanding of the expected effects of healthcare innovations. Participatory health economic modelling is an important tool to support the design of complex interventions with increased levels of diffuse, variable and spillover effects. Involving stakeholders in the modelling process is important because it enables stakeholders within complex-adaptive systems to form a shared understanding of options, probabilities and impacts. The appropriate representation of uncertainty is critical to the validity and value of early health economic modelling, including parameter and structural uncertainty.

## Acknowledgements

Funding from the Australian National Health and Medical Research Council (NHMRC) Partnership Centre for Health System Sustainability (Grant ID: 9100002) supported the drafting of this manuscript.

## Ethical issues

Not applicable.

## Competing interests

Authors declare that they have no competing interests.

## Authors’ contributions

Following discussions between the authors, AP wrote the first draft of the manuscript, followed by an iterative process of editing and commenting.

## Authors’ affiliations

^1^Flinders Health and Medical Research Institute, Flinders University, Adelaide, SA, Australia. ^2^Macquarie University Centre for the Health Economy, Macquarie University, Macquarie Park, NSW, Australia.
